# The clinical profile, genetic basis and survival of childhood cardiomyopathy: a single-center retrospective study

**DOI:** 10.1007/s00431-023-05358-6

**Published:** 2024-01-02

**Authors:** Wenjing Yuan, Zhongli Jia, Jiajin Li, Lingjuan Liu, Jie Tian, Xupei Huang, Junjun Quan

**Affiliations:** 1 Department of Cardiology, Ministry of Education Key Laboratory of Child Development and Disorders, National Clinical Research Center for Child Health and Disorders, China International Science and Technology Cooperation Base of Child Development and Critical Disorders, Chongqing Key Laboratory of Pediatrics, National Clinical Key Cardiovascular Specialty, Children’s Hospital of Chongqing Medical University, 136 Zhongshan Er Road, Yu Zhong District, Chongqing, 400014 People’s Republic of China; 2 Department of Biomedical Science, Charles E. Schmidt College of Medicine, Florida Atlantic University, Boca Raton, FL USA

**Keywords:** Cardiomyopathy, Children, Clinical characteristics, Outcome, Risk factor

## Abstract

Cardiomyopathy (CM) is a heterogeneous group of myocardial diseases in children. This study aimed to identify demographic features, clinical presentation and prognosis of children with CM. Clinical characteristics and prognostic factors associated with mortality were evaluated by Cox proportional hazards regression analyses. Genetic testing was also conducted on a portion of patients. Among the 317 patients, 40.1%, 25.2%, 24.6% and 10.1% were diagnosed with dilated cardiomyopathy (DCM), hypertrophic cardiomyopathy (HCM), left ventricular noncompaction cardiomyopathy (LVNC) and restrictive cardiomyopathy (RCM), respectively. The most common symptom observed was dyspnea (84.2%). Except for HCM, the majority of patients were classified as NYHA/Ross class III or IV. The five-year survival rates were 75.5%, 67.3%, 74.1% and 51.1% in DCM, HCM, LVNC and RCM, respectively. The ten-year survival rates were 60.1%, 56.1%, 57.2% and 41.3% in DCM, HCM, LVNC and RCM, respectively. Survival was inversely related to NYHA/Ross class III or IV in patients with DCM, HCM and RCM. Out of 42 patients, 32 were reported to carry gene mutations.

*Conclusions*: This study demonstrates that CM, especially RCM, is related to a high incidence of death. NYHA/Ross class III or IV is a predictor of mortality in the patients and gene mutations may be a common cause.

*Trial registration*: MR-50-23-011798.

**Table:** 

**What is Known:** *• Cardiomyopathy (CM) is a heterogeneous group of myocardial diseases and one of the leading causes of heart failure in children due to the lack of effective treatments.* *• There remains scarce data on Asian pediatric populations though emerging studies have assessed the clinical characteristics and outcomes of CM.*	
**What is New:** *• A retrospective study was conducted and the follow-up records were established to investigate the clinical characteristics, the profile of gene mutations and prognostic outcomes of children with CM in Western China.* *• CM, especially RCM, is related to a high incidence of death. NYHA/Ross class III or IV is a predictor of mortality in the patients and gene mutations may be a common cause.*

**Supplementary Information:**

The online version contains supplementary material available at 10.1007/s00431-023-05358-6.

## Introduction

Pediatric cardiomyopathy (CM) is a heterogeneous group of myocardial diseases and one of the leading causes of heart failure (HF) in children due to the lack of effective treatments [1]. The overall prevalence of CM in children is 2.11/100,000 [2]. Based on hemodynamical and morphological features, pediatric cardiomyopathies are mainly divided into four subtypes, dilated cardiomyopathy (DCM), hypertrophic cardiomyopathy (HCM), restrictive cardiomyopathy (RCM), and unclassified cardiomyopathy such as left ventricular noncompaction cardiomyopathy (LVNC). Unfortunately, the symptoms and signs among pediatric cardiomyopathies are non-specific and similar. As a result, early diagnosis and treatment are often difficult.

In recent decades, emerging studies have assessed the clinical progression of CM, Nevertheless, there remains a scarcity of data on Asian populations [3, 4]. The clinical features and profile of CM in the Chinese population may differ from those in other ethnic groups. CM is not yet fully comprehended because of its infrequent occurrence, particularly among children. Our study was sought to investigate the clinical characteristics and prognostic outcomes of children with CM in Western China, to identify risk factors for CM-related death and to analyze the profile of gene mutations in this group.

## Methods

### Patients and data collection

A retrospective study was approved by the ethics committee of Children’s Hospital of Chongqing Medical University (Chongqing, China) and conducted at the hospital. Out of the potential 367 cases responded to the study survey, a total of 317 patients (168 males, 149 females) ≤ 18 years old diagnosed respectively with DCM, HCM, LVNC, or RCM between November 2007 and May 2023 at Children’s Hospital of Chongqing Medical University, were included in this study.

Eligible participants amongst patients with CM were limited to those who displayed inherent myocardial abnormalities. The inclusion criteria for DCM were left ventricular dilation (left ventricular end diastolic diameter ≥ 2 SD above normal for body-surface area), simultaneously reduced left ventricular systolic function (left ventricular ejection fraction (LVEF) ≤ 2 SD below normal for age), and an absence of secondary causes of ventricular dilation [5]. Diagnosis of HCM was based on left ventricular hypertrophy (a wall thickness ≥ 2 SD above the normal population mean for body surface area), excluding defined hemodynamic causes such as congenital heart disease, hypertension, or exposure to drugs known to result in cardiac hypertrophy [6]. Meanwhile, RCM patients included children who exhibited enlarged atria without ventricular dilatation in the absence of congenital, valvular, or pericardial disease [7]. Finally, patients were diagnosed with LVNC as defined by the Jenni criteria [8].

Data, including demographic profile and clinical information associated with CM, were collected for all patients based on their electronic medical records or clinical charts. Z-score of echocardiographic parameters was established by http://hdb.nbscn.org/zscore. New York Heart Association (NYHA) class or Ross functional Class, detailed ECG and echocardiography were also reviewed. For CM patients who were admitted to the hospital in recent years, informed consent was obtained and peripheral blood samples were collected for next generation sequencing. Follow-up data were gathered through telephone call or at clinic visit. Since data analyzed were on retrospective cases of patients who had received standard diagnosis and therapy, informed consent from some patients was not obtained.

Follow-up records were established for all patients, and telephone follow-up were used once a year after the first hospitalization and discharge for a total of 10 years. The exercise tolerance and survival time of the children were recorded. The end point of follow-up was cardiac death.

### Statistical analysis

Statistical analyses were performed using SPSS Statistics Version 23.0 (IBM Corporation, Armonk, USA). Continuous variables of normal distribution were expressed as mean ± standard deviation and compared by ANOVA followed by Tukey’s or Bonferoni’s or Sidak’s multiple comparison post hoc. Skewed distributions are described with medians and interquartile ranges (IQR). While categorical variables were expressed as frequency (percentage) and analyzed by Chi square test or Fisher's exact test. Survival follow-up data were analyzed with Kaplan-Meier curve followed by a log-rank test for significance. The variables associated with CM or HF were analyzed by univariate Cox proportional hazards repression analyses with predictors of mortality and 95% confidence interval (95% CI). Variables with a probability value of < 0.05 in univariate analyses were candidates for multivariable Cox proportional hazards models. *P* value < 0.05 was considered statistically significant.

## Results

### Baseline profile and clinical characteristics

The baseline profile and clinical characteristics of the four CM groups are summarized in Table 1. Among the 317 patients (168 boys, 53%), DCM was diagnosed in 127 cases (40.1%, 65 boys and 62 girls), HCM in 80 cases (25.2%, 50 boys and 30 girls), LVNC in 78 cases (24.6%, 34 boys and 44 girls), and RCM in 32 cases (10.1%, 19 boys and 13 girls) (Supplemental Fig. 1 and Table 1). The age at diagnosis of CM differed significantly among the four groups (*P* < 0.001) (Supplemental Fig. 2). Age at diagnosis of DCM was significantly higher than HCM [6.5 (3.5, 10.1) years VS 0.5 (0.3, 1.8) years, *P* < 0.001] and LVNC [6.5 (3.5, 10.1) years VS 0.8 (0.3, 3.7) years, *P* < 0.001], but not when compared with RCM [6.5 (3.5, 10.1) years VS 4.9 (1.9, 8.7) years, *P* > 0.05].

**Table 1:** Baseline profile and clinical characteristics of the study population

	**Total**	**DCM**	**HCM**	**LVNC**	**RCM**
	**(n = 317)**	**(n = 127)**	**(n = 80)**	**(n = 78)**	**(n = 32)**
Age (year)	4.5 ± 4.4	6.8 ± 4.1	2.4 ± 4^a^	2.6 ± 3.4^a^	5.3 ± 4^bc^
Sex (male)	168 (53%)	65 (51.2%)	50 (62.5%)	34 (43.6%)^b^	19 (59.4%)
Family history	11 (3.5%)	5 (3.9%)	3 (3.8%)	1 (1.3%)	2 (6.3%)
Pneumonia	151 (47.6%)	40 (31.5%)	50 (62.5%)^a^	43 (55.1%)^a^	18 (56.3%)^a^
Cyanosis	185 (58.4%)	59 (46.5%)	55 (68.8%)^a^	49 (62.8%)^a^	22 (68.8%)^a^
Dyspnea	267 (84.2%)	112 (88.2%)	64 (80%)	63 (80.8%)	28 (87.5%)
Syncope	23 (7.3%)	10 (7.9%)	9 (11.3%)	3 (3.8%)	1 (3.1%)
Cardiomegaly	161 (50.8%)	86 (67.7%)	19 (23.8%)^a^	36 (46.2%)^b^	20 (62.5%)^b^
Cardiac murmur	137 (43.2%)	42 (33.1%)	41 (51.3%)^a^	38 (48.7%)^a^	16 (50%)
Jugular venous distension	58 (18.3%)	33 (26%)	2 (2.5%)^a^	9 (11.5%)^ab^	14 (43.8%)^bc^
Hepatomegaly	188 (59.3%)	102 (80.3%)	29 (36.3%)^a^	29 (37.2%)^a^	28 (87.5%)^bc^
Orthopnoea	47 (14.8%)	30 (23.6%)	2 (2.5%)^a^	8 (10.3%)^ab^	7 (21.9%)^b^
Peripheral edema	118 (37.2%)	80 (63%)	7 (8.8%)^a^	12 (15.4%)^a^	19 (59.4%)^bc^
NYHA /Ross class					
I	40 (12.6%)	10 (7.9%)	20 (25%)	9 (11.5%)^a^	1 (3.1%)
II	103 (32.5%)	38 (29.9%)	35 (43.8%)^a^	23 (29.5%)	7 (21.9%)^b^
III	96 (30.3%)	44 (34.6%)	18 (22.5%)	23 (29.5%)	11 (34.4%)
IV	78 (24.6%)	35 (27.6%)	7 (8.8%)^a^	23 (29.5%)^b^	13 (40.6%)^b^
Basic medication					
Digoxin	148 (46.7%)	96 (75.6%)	0 (0%)	52 (66.7%)	0 (0%)
β-blockers	64 (20.2%)	35 (27.6%)	20 (25%)	6 (7.7%)^ab^	3 (9.4%)^ab^
Diuretics	204 (64.4%)	106 (83.5%)	19 (23.8%)^a^	57 (73.1%)^b^	22 (68.8%)^b^
Aspirin	44 (13.9%)	13 (10.2%)	1 (1.3%)^a^	27 (34.6%)^ab^	3 (9.4%)^c^
ACEI/ARBs	187 (59%)	98 (77.2%)	21 (26.3%)^a^	56 (71.8%)^b^	12 (37.5%)^ac^
Calcium channel blockers	4 (1.3%)	2 (1.6%)	2 (2.5%)	0 (0%)	0 (0%)
Amiodarone	8 (2.5%)	8 (6.3%)	0 (0%)	0 (0%)	0 (0%)
ECG data					
Premature atrial contraction	14 (4.4%)	11 (8.7%)	1 (1.3%)^a^	1 (1.3%)^a^	1 (3.1%)
Premature ventricular contraction	29 (9.1%)	19 (15%)	3 (3.8%)^a^	7 (9%)	0 (0%)
Atrial tachycardia	14 (4.4%)	11 (8.7%)	3 (3.8%)	0 (0%)	0 (0%)
Supraventricular tachycardia	5 (1.6%)	5 (3.9%)	0 (0%)	0 (0%)	0 (0%)
Ventricular tachycardia	14 (4.4%)	9 (7.1%)	1 (1.3%)	4 (5.1%)	0 (0%)
Bundle branch block	17 (5.4%)	8 (6.3%)	2 (2.5%)	5 (6.4%)	2 (6.3%)
Atrioventricular block	26 (8.2%)	18 (14.2%)	1 (1.3%)	6 (7.7%)	1 (1.3%)
Wolff-Parkinson-White syndrome	11 (3.5%)	6 (4.7%)	2 (2.5%)	3 (3.8%)	0 (0%)
ST-T wave changes	4 (1.3%)	4 (3.2%)	0 (0%)	0 (0%)	0 (0%)
Heart rate (bpm)	115.8 ± 24.5	115.2 ± 23.4	118.5 ± 29	121.5 ± 19.2^a^	97.4 ± 19.2^abc^
P (ms)	82.5 ± 24.5	84.8 ± 27.1	74.9 ± 20.4	80.1 ± 23.6	98.2 ± 15.8^abc^
P-R (ms)	126.5 ± 32.8	135 ± 34.7	110.2 ± 28^a^	126.9 ± 32.5	131.9 ± 20.9^b^
QT (ms)	311.3 ± 49	316.9 ± 50.8	307.3 ± 51.9	298.2 ± 35.4^a^	331.1 ± 54.9^c^
QTc (ms)	423.9 ± 53.2	435.4 ± 51.4	414.1 ± 63.7^a^	422.7 ± 39.2	405.2 ± 53.1^a^
QRS (ms)	89.7 ± 19.2	90.5 ± 19.5	87.1 ± 21.6	93.1 ± 17.4	85 ± 14.3
Echocardiographic data					
EF	50.1 ± 21.4	39.5 ± 10.1	64.7 ± 13.3^a^	44.7 ± 14.9^ab^	60.5 ± 9.4^ac^
FS	25.1 ± 11	19 ± 5.1	34.5 ± 12.8^a^	22.5 ± 9.3^ab^	32 ± 6.5^ac^
IVRT (ms)	74.8 ± 29.4	75.3 ± 28.7	76.5 ± 33.5	66.2 ± 24.2^a^	89.6 ± 26.9^abc^
E/A	1.5 ± 0.6	1.7 ± 0.5	1.1 ± 0.5^a^	1.5 ± 0.5^ab^	2.1 ± 0.6^abc^
LVEDD (mm)	40.2 ± 14.8	52.1 ± 9.7	24.2 ± 7.4^a^	40.3 ± 11.8^ab^	33 ± 7.8^abc^
LVEDD-Z-score	4.4 ± 5.2	7.3 ± 3.6	-1.1 ± 2.6*	6.6 ± 4.7^b^	-0.1 ± 2.4^ac^
RVEDD (mm)	13.4 ± 5	14.8 ± 4.4	10.5 ± 3.7^a^	14.6 ± 6^b^	12.6 ± 3.4^abc^
LVPW (mm)	/	/	8.6 ± 4.3	/	/
LVPW-Z-score	/	/	5.6 ± 6.4	/	/
IVS (mm)	/	/	11.5 ± 6.4	/	/
N/C	/	/	/	2.2 ± 0.6	/
LA (mm)	/	/	/	/	33.7 ± 6.9
RA (mm)	/	/	/	/	33.7 ± 6.6
Outflow obstruction	/	/	23 (28.8%)	/	/
Mitral regurgitation (moderate-severe)	97 (30.6%)	63 (49.6%)	5 (6.3%)^a^	14 (17.9%)^ab^	15 (46.9%)^bc^
Tricuspid regurgitation (moderate-severe)	58 (18.3%)	29 (22.8%)	1 (1.3%)^a^	14 (17.9%)^b^	14 (43.8%)^abc^
Pericardial effusion	77 (24.3%)	36 (28.3%)	15 (18.8%)	9 (11.5%)	17 (53.1%)

Continuous data are presented as mean ± SD and categorical variables are presented as number (percent). Values are expressed as means ± SD. Statistical significance was determined by ANOVA followed by Tukey’s or Bonferoni’s or Sidak’s multiple comparison post hoc

*DCM* dilated cardiomyopathy, *E/A* early mitral inflow E to late mitral inflow A ratio, *EF* ejection fraction *FS* fractional shortening of LV, *HCM* hypertrophic cardiomyopathy, *IVRT* isovolumetric relaxation time, *IVS* intraventricular septum, *LA* left atria, *LVEDD* left ventricle end diastolic dimension, *LVNC* left ventricular noncompaction cardiomyopathy, *LVPW* left ventricle posterior wall, *N/C* noncompacted to compacted ratio, *RA* right atria, *RCM* restrictive cardiomyopathy, *RVEDD* right ventricle end diastolic dimension

^a^*P* < 0.05 compared to DCM

^b^*P* < 0.05 compared to HCM

^c^*P* < 0.05compared to LVNC

Of all groups, 11 patients (3.5%) exhibited positive family history, with 5 cases of DCM, 3 cases of HCM, 1 patient with LVNC, and two RCM patients. The initial symptoms of CM patients were nonspecific and somewhat varied upon diagnosis. The prevalent symptom among all patients was dyspnea, followed by hepatomegaly, cyanosis, and cardiomegaly. The proportions of NYHA/Ross class of I, II, III, IV in all CM patients were 40 (12.6%), 103 (32.5%), 96 (30.3%) and 78 (24.6%) patients, respectively. Except for HCM, the majority of patients were classified as NYHA/Ross class III or IV. Diuretics were frequently prescribed to in 204 (64.4%) patients. 134 patients (65.4%) in the DCM and LVNC groups received treatment with digoxin and ACEI and/or ARBs. Nearly one in five patients with DCM and HCM received β-blockers, whilst aspirin was more widely used in patients with LVNC as compared to the other three groups. Eight DCM patients were prescribed amiodarone due to tachycardia.

### Electrocardiography

Nonspecific ECG abnormalities were present in 111 patients (35%) with CM (Table 1). The most prevalent was premature ventricular contraction, followed by atrioventricular blockade, bundle branch blockade, premature atrial contraction, atrial tachycardia, ventricular tachycardia, and Wolff-Parkinson-White (WPW) syndrome. All ECG abnormalities were found to be more prevalent among DCM patients. Patients with LVNC were more likely to develop arrhythmias with definite or potential effects on hemodynamics, compared to patients with HCM or RCM. Meanwhile, P wave of RCM patients was greater than those of other CM patients. QTc intervals in RCM patients were shorter than those in other type of CM patients.

### Echocardiography

Echocardiographic data on all CM patients at initial presentation are summarized in Table 1. Left ventricular ejection fraction (EF) and fractional shortening (FS) were significantly decreased in patients with DCM and LVNC (39.5% vs. 44.7%, 19% vs. 22.5%, respectively), and systolic function was worse in patients with DCM (*P* < 0.05). Consistent with systolic function, left ventricular end-diastolic diameter (LVEDD) and right ventricular end-diastolic diameter (RVEDD) increased in the DCM and LVNC groups (52.1 mm vs. 40.3 mm, 14.8 mm vs. 14.6 mm, respectively). The isovolumic relaxation time (IVRT) in patients with RCM was significantly longer than others. 24 patients (75%) had a significantly prolonged IVRT of over 80 ms. IVRT in HCM was 76.5 ± 33.5 ms, and in 43 patients (53.8%) exceeded 80 ms. The mean noncompacted to compacted (N/C) ratio was 2.2 in patients with LVNC, 39 patients demonstrated N/C ratio > 2.

Severe valvular regurgitation and pericardial effusion were prevalent in cardiomyopathy. Nearly half of patients with RCM progressive severe mitral and tricuspid regurgitation, as well as pericardial effusion. Mitral regurgitation was the most common valvular regurgitation in DCM, and around a quarter of patients had pericardial effusion. They were less common in HCM compared to other types of cardiomyopathies.

### Clinical course

**Fig. 1:**
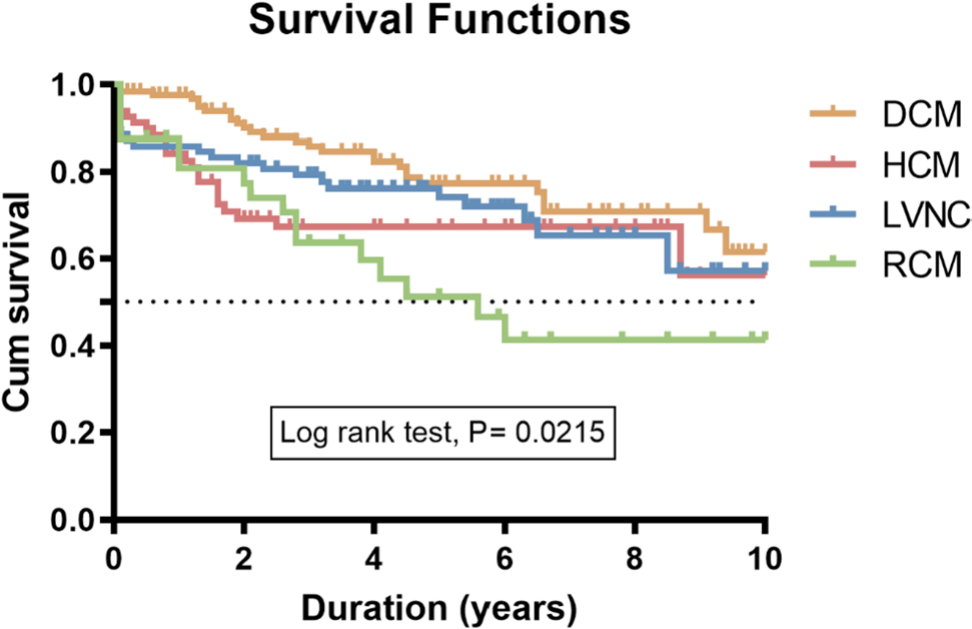
Kaplan-Meier survival curves for the subgroups of cardiomyopathy and log-rank test for cumulative survival rates (*P* = 0.0215). DCM, dilated cardiomyopathy; HCM, hypertrophic cardiomyopathy; LVNC, left ventricular noncompaction cardiomyopathy; RCM, restrictive cardiomyopathy

The cumulative survival rates among the four groups were statistically different (log-rank *P* = 0.0215) according to Kaplan-Meier survival analysis, as shown in Fig. 1. The five-year survival rates were 75.5%, 67.3%, 74.1% and 51.1% in DCM, HCM, LVNC and RCM, respectively. The ten-year survival rates were 60.1%, 56.1%, 57.2% and 41.3% in DCM, HCM, LVNC and RCM, respectively. The mean survivals were 7.8 (95% CI, 7.2–8.5), 6.9 (95% CI, 5.9–7.9), 7.3 (95% CI, 6.4–8.2) and 5.7 (95% CI, 4.3–7.2) years in DCM, HCM, LVNC, and RCM, respectively. At follow-up, 92 (29%) patients with CM died, including 30 (23.6%) DCM, 23 (28.8%) HCM, 23 (29.5%) LVNC and 16 (50%) RCM patients. RCM patients demonstrated the highest mortality and the lowest survival rate compared to the other three groups of CM patients.

As shown in Tables 2 and 3, the models used in proportional hazard analysis included multiple variables, which corresponded to the type of cardiomyopathies. Based on univariate analysis on the DCM group, age, sex, family history, symptoms, and most of the electrocardiographic and echocardiographic parameters were not related to mortality. However, the risk of death was significantly associated with NYHA/Ross class III or IV, QTc interval, FS, and moderate-severe TR. Meanwhile, multivariate analysis demonstrated that the risk of death was associated with NYHA/Ross class III or IV (HR = 3.77, 95% CI = 1.3–10.9, *P* = 0.014), but not related to QTc interval, EF, nor FS. Although LVNC patients showed similar clinical manifestations to patients with DCM, univariate analysis demonstrated that NYHA/Ross class, EF, FS, QTc interval, and moderate-severe TR were not related to outcomes. In addition, age, sex, and family history were not predictors for LVNC survival. However, results of univariate analysis indicated that QRS duration, RVEDD, and N/C ratio were risk factors for LVNC. Based on multivariate analysis, survival was not significantly associated with QRS duration or N/C ratio, but significant association was observed with RVEDD (HR = 1.06, 95% CI = 1–1.11, *P* = 0.046).

**Table 2:** Univariate analysis of risk factors for death

	**DCM**		**HCM**		**LVNC**		**RCM**	
	**HR (95%CI)**	***P* value**	**HR (95%CI)**	***P* value**	**HR (95%CI)**	***P* value**	**HR (95%CI)**	***P* value**
Age (year)	1.02 (0.93–1.11)	0.707	0.91 (0.79–1.05)	0.193	1 (0.88–1.13)	0.947	0.88 (0.76–1.01)	0.070
< 1 year	0.05 (0.01–61.11)	0.401	2.25 (0.76–6.65)	0.141	1.53 (0.66–3.55)	0.322	2.67 (0.75–9.51)	0.131
Sex (male)	1.24 (0.6–2.56)	0.561	0.98 (0.41–2.32)	0.959	1.51 (0.67–3.43)	0.324	3.55 (1.01–12.51)	0.049
Family history	1.23 (0.17–9.11)	0.836	1.14 (0.15–8.5)	0.897	0.76 (0–8.75)	0.620	8.8 (1.6–48.55)	0.013
Pneumonia	0.77 (0.34–1.72)	0.518	1.73 (0.68–4.43)	0.253	1.21 (0.53–2.77)	0.649	2.58 (0.88–7.54)	0.084
Cyanosis	1.18 (0.57–2.41)	0.659	2.18 (0.74–6.43)	0.158	1.77 (0.7–4.51)	0.229	1.88 (0.54–6.62)	0.323
Dyspnea	1.84 (0.44–7.75)	0.405	2.48 (0.58–10.6)	0.219	1.8 (0.53–6.08)	0.344	24.13 (0.82–2.43)	0.367
Syncope	1.56 (0.47–5.21)	0.469	0.75 (0.18–3.21)	0.699	0.98 (0–2.33)	0.479	1.6 (0.21–12.33)	0.651
Cardiomegaly	0.36 (0.08–1.54)	0.169	2.22 (0.75–6.55)	0.149	1.4 (0.33–5.99)	0.650	22.91 (0.81–6.68)	0.442
Cardiac murmur	1.08 (0.5–2.31)	0.848	0.55 (0.23–1.29)	0.167	1.06 (0.47–2.41)	0.887	0.96 (0.36–2.56)	0.934
Jugular venous distension	1.66 (0.8–3.45)	0.176	6.07 (1.37–26.82)	0.017	1.09 (0.32–3.66)	0.894	0.52 (0.19–1.46)	0.215
Hepatomegaly	1.06 (0.4–2.77)	0.910	3.63 (1.52–8.67)	0.004	1.25 (0.55–2.86)	0.593	0.44 (0.12–1.57)	0.205
Orthopnoea	1.83 (0.87–3.86)	0.112	0.75 (0.66–10.33)	0.724	0.72 (0.17–3.07)	0.652	2.82 (1.02–7.83)	0.047
NYHA/Ross class III or IV	4.57 (1.6–13.11)	0.005	2.52 (1.11–5.73)	0.028	0.82 (0.34–2)	0.664	2.06 (1.04–4.07)	0.037
Heart rate	0.99 (0.98–1.01)	0.328	1 (0.99–1.02)	0.849	1.02 (1–1.04)	0.128	1.03 (1–1.06)	0.068
P	1 (0.99–1.02)	0.664	0.99 (0.97–1.01)	0.441	1.01 (0.99–1.02)	0.389	1 (0.97–1.03)	0.804
P-R	1 (0.99–1.01)	0.940	0.97 (0.95–0.99)	0.002	1.01 (1–1.01)	0.060	0.97 (0.95–1)	0.024
QT	1 (0.99–1.01)	0.632	1 (0.99–1)	0.314	1 (0.99–1.02)	0.518	1 (0.99–1.01)	0.493
QTc	1.03 (0.98–1.04)	0.015	1 (0.99–1.01)	0.919	1.01 (1–1.02)	0.070	1 (0.99–1)	0.338
QRS	1 (0.99–1.02)	0.612	1.02 (0.99–1.04)	0.158	1.03 (1–1.05)	0.027	1 (0.97–1.03)	0.916
EF	0.92 (0.88–0.97)	0.001	0.96 (0.93–1)	0.031	1 (0.97–1.03)	0.962	0.95 (0.9–1)	0.058
FS	0.88 (0.8–0.96)	0.004	0.98 (0.95–1.02)	0.319	0.99 (0.94–1.04)	0.597	0.94 (0.88–1.02)	0.120
IVRT	1 (0.99–1.01)	0.794	1 (0.98–1.01)	0.580	1 (0.99–1.02)	0.611	0.98 (0.96–1)	0.076
E/A	1.09 (0.55–2.17)	0.810	0.73 (0.33–1.6)	0.429	1.19 (0.56–2.54)	0.656	0.65 (0.29–1.46)	0.300
LVEDD	1.01 (0.97–1.05)	0.663	1.02 (0.96–1.08)	0.550	0.99 (0.95–1.03)	0.506	0.94 (0.88–1)	0.044
LVEDD-Z-score	0.98 (0.9–1.08)	0.494	0.99 (0.89–1.11)	0.984	0.94 (0.88–1.04)	0.065	0.76 (0.59–98)	0.032
RVEDD	1.03 (0.95–1.11)	0.457	1.04 (0.96–1.13)	0.341	1.06 (1–1.12)	0.033	1.07 (0.9–1.28)	0.428
LVPW	/	/	1.12 (1.03–1.22)	0.006	/	/	/	/
LVPW-Z-score	/	/	1 (0.99–1.05)	0.726	/	/	/	/
IVS	/	/	1.03 (0.98–1.08)	0.276	/	/	/	/
N/C	/	/	/	/	1.73 (1.02–2.92)	0.041	/	/
LA	/	/	/	/	/	/	0.98 (0.91–1.05)	0.563
RA	/	/	/	/	/	/	0.96 (0.89–1.03)	0.269
Outflow obstruction	/	/	0.82 (0.32–2.08)	0.679	/	/	/	/
Mitral regurgitation (moderate-severe)	1.03 (0.5–2.12)	0.931	0.8 (0.11–5.95)	0.826	0.66 (0.19–2.22)	0.500	1.64 (0.61–4.39)	0.327
Tricuspid regurgitation (moderate-severe)	2.19 (1.01–4.75)	0.047	0.75 (0.34–9.9)	0.782	1.34 (0.5–3.62)	0.562	0.89 (0.32–2.45)	0.815
Pericardial effusion	1.85 (0.89–3.85)	0.100	2.62 (1.06–6.44)	0.036	0.31 (0.042–2.338)	0.258	3.05 (0.981–9.505)	0.054
Premature atrial contraction	0.67 (0.16–2.81)	0.582	6.35 (0.82–49.25)	0.077	3.25 (0.43–24.38)	0.251	0.45 (0.95–7.68)	0.533
Premature ventricular contraction	2.08 (0.88–4.89)	0.094	0.98 (0.86–6.21)	0.527	1.18 (0.27–5.08)	0.824		
Atrial tachycardia	0.6 (0.14–2.54)	0.491	1.37 (0.18–10.18)	0.761				
Ventricular tachycardia	1.74 (0.53–5.75)	0.365	0.89 (0.55–7.02)	0.676	0.45 (0.134–115.69)	0.439		
Supraventricular tachycardia	0.72 (0.09–5.29)	0.744						
Bundle branch block	0.91 (0.22–3.82)	0.898	6.72 (0.82–55.04)	0.076	1.7 (0.4–7.26)	0.477	0.91 (0.12–6.94)	0.929
Atrioventricular block	0.99 (0.37–2.62)	0.982			2.01 (0.61–6.95)	0.247	1.83 (0.24–14.19)	0.561
Wolff-Parkinson-White syndrome	0.55 (0.17–1.91)	0.563	4.97 (1.15–21.37)	0.031	1.05 (0.14–7.91)	0.959		

*DCM* dilated cardiomyopathy, *E/A* early mitral inflow E to late mitral inflow A ratio, *EF* ejection fraction, *FS* fractional shortening of Left ventricle, *HCM* hypertrophic cardiomyopathy, *IVRT* isovolumetric relaxation time, *IVS* intraventricular septum, *LA* left atria, *LVEDD* left ventricle end diastolic dimension, *LVNC* left ventricular noncompaction cardiomyopathy, *LVPW* left ventricle posterior wall, *N/C* noncompacted to compacted ratio, *RA* right atria, *RCM* restrictive cardiomyopathy, *RVEDD* right ventricle end diastolic dimension

**Table 3:** Multivariate analysis of risk factors for death

	**HR**	**(95%CI)**	***P* value**
DCM			
NYHA/Ross class III or IV	3.8	(1.3–10.9)	0.014
QTc	1	(1.01–10.9)	0.044
EF	0.9	(0.8–1.05)	0.208
FS	1	(0.81–1.34)	0.74
Tricuspid regurgitation	1.7	(0.76–3.62)	0.204
HCM			
Jugular venous distension	2	(0.29–13.03)	0.491
Hepatomegaly	2.7	(1–7.4)	0.05
NYHA/Ross class III or IV	1.3	(0.49–3.39)	0.612
P-R	1	(0.96–1)	0.096
EF	1	(0.95–1.03)	0.501
LVPW	1.1	(0.94–1.17)	0.398
Pericardial effusion	1.5	(0.54–4.43)	0.424
Wolff-Parkinson-White syndrome	3.7	(0.59–22.99)	0.165
LVNC			
QRS	1	(1–1.05)	0.077
RVEDD	1.1	(1–1.11)	0.046
N/C	1.3	(0.77–2.34)	0.295
RCM			
Male	5.4	(1.08–26.99)	0.04
Family history	3.4	(0.37–31.02)	0.277
Orthopnoea	1.3	(0.34–5.1)	0.681
NYHA/Ross class III or IV	8.7	(1.02–73.43)	0.048
P-R	1	(0.97–1.05)	0.719
LVEDD	0.9	(0.82–0.98)	0.018
LVEDD-Z Score	1	(0.778–1.286)	0.029

*DCM* dilated cardiomyopathy, *EF* ejection fraction, *FS* fractional shortening of Left ventricle, *HCM* hypertrophic cardiomyopathy, *LVPW* left ventricle posterior wall, *LVNC* left ventricular noncompaction cardiomyopathy, *RVEDD* right ventricle end diastolic dimension, *N/C* noncompacted to compacted ratio, *RCM* restrictive cardiomyopathy, *LVEDD* left ventricle end diastolic dimension

In HCM patients, univariate Cox repression analysis identified eight predictors of survival: jugular venous distension, hepatomegaly, NYHA/Ross class III or IV, P-R interval, EF, LVPW thickness, pericardial effusion, and WPW syndrome. Based on results from multivariate analysis, these eight factors did not show significant association with death. In addition, no positive indications that age, sex, family history, syncope, and IVS thickness contributed to death were detected in this study. Univariate Cox repression analysis on RCM patients showed that the predictor of survival was related to male sex, family history, orthopnea, NYHA/Ross class III or IV, P-R interval, and LVEDD. Meanwhile, age, IVRT, E/A ratio, LA diameter and RA diameter did not show a statistical association with mortality. Furthermore, the multivariate-adjusted analysis of significant variables described above demonstrated that male sex (HR = 5.41, 95% CI = 1.08–26.99, *P* = 0.040) and NYHA/Ross class III or IV (HR = 8.65, 95% CI = 1.02–73.43, *P* = 0.048) were independent risk factors for the prediction of death in patients with RCM, while high LVEDD (HR = 0.90, 95% CI = 0.82–0.98, *P* = 0.018) was a protective factor.

### Genetic analysis

Mutation analysis of CM was performed in 42 patients, along with a portion of data reported in our previous study, as shown in Table 4. Overall, gene mutations were identified in 32 (76.2%) children with CM, a total of 14 pathogenic mutations were found, 9 mutations were likely pathogenic and 24 mutations were variants of uncertain significance. Meanwhile, the parents of 19 individuals (45.2%) had positive findings in next-generation sequencing. Out of 15 RCM patients analyzed, 8 subjects carried gene mutations. Mutations at site of 192 (Arg192Cys and Arg192His) in *TNNI3* were identified in 3 patients. In addition, 12 (80.0%) RCM children died, exhibiting the worst prognosis, including three patients with multi-gene mutations or compound heterozygous mutations. Of 18 patients with HCM, all carried gene mutations. Of these patients, 6 carried multi-gene mutations. *MYH7* gene variants were most frequently observed and were found in 6 of the children with HCM. Compound heterozygous mutations (Ile30670Thr and Arg24331His) in *TTN* were novel and were detected in only one HCM children. Five children with GAA mutations were admitted with significant cardiac hypertrophy by echocardiography and were finally diagnosed with secondary HCM (Pompe Disease). Age at diagnosis was less than one year in eight of the 12 patients with HCM who died. Mutations in *MYH7* and myopalladin (*MYPN*) were identified in one of three children with LVNC.

**Table 4:** Genetic analysis of partial patients

**Patient No.**	**Sex**	**Diagnosis**	**Age at initial presentation (yrs)**	**Gene**	**Mutation**	**Variant type**	**Carrier**	**Status**
1	M	RCM	8.2	*TNNI3*	Arg192Cys	P	None	Alive
2	F	RCM	5.5	*TNNI3* *PKP2*	Arg192His Ala749Asp	P P	None None	Died
3	F	RCM	1.6	*TNNI3* *MYBPC3*	Arg192Cys Arg1002Trp	P LP	Mother Mother	Died
4	F	RCM	2	*DSP* *DSP* *ILK*	Gln1648Arg Arg2075Trp Asn236Ser	VUS VUS VUS	Father Mother Mother	Died
5	F	RCM	7.6	*MYH7*	Leu863Pro	LP	None	Died
6	M	RCM	10.7	*MYH7*	Splicing	VUS	None	Died
7	M	RCM	14.1	*MYH7*	Splicing	VUS	Father	Died
8	M	RCM (Salih myopathy)	5.0	*TTN*	Asn17193Ser	VUS	None	Alive
9	F	RCM	9.5	Undetected	/	/	/	Died
10	F	RCM	4.2	Undetected	/	/	/	Died
11	M	RCM	2.7	Undetected	/	/	/	Died
12	F	RCM	1.5	Undetected	/	/	/	Died
13	F	RCM	7.5	Undetected	/	/	/	Died
14	M	RCM	6.8	Undetected	/	/	/	Alive
15	M	RCM	0.8	Undetected	/	/	/	Died
16	M	HCM	8.2	*MYH7*	Ala254Glu	VUS	None	Died
17	M	HCM	12.8	*MYH7*	Met822Val	P	None	Alive
18	F	HCM	3.4	*MYH7*	Thr441Met	P	None	Died
19	F	HCM	7	*MYH7*	Leu863Pro	LP	None	Alive
20	M	HCM	0.1	*MYH7* *MYBPC3*	Glu924Lys Met555Thr	LP LP	Mother Mother	Alive
21	M	HCM	9.5	*MYH6* *RAF1*	Arg1214Trp Ser259Thr	VUS P	Mother None	Alive
22	F	HCM	0.5	*TPM1* *RAF1*	Splicing Ser257Leu	VUS P	Mother None	Died
23	F	HCM	0.8	*TTN* *TTN*	Ile30670Thr Arg24331His	VUS VUS	Mother Father	Died
24	F	HCM	0.5	*TTN* *MYL2*	Arg6136Gln Met36Val	VUS VUS	Mother Mother	Died
25	M	HCM	0.9	*NEXN*	Arg279Cys	VUS	Father	Died
26	F	HCM	13.5	*TNNI3*	Arg186Gln	P	None	Died
27	F	HCM (Pompe Disease)	0.5	*GAA*	Arg672Gln	P	Parents	Alive
28	F	HCM (Pompe Disease)	0.5	*G6PC* *GAA*	Val124Met Cys103Arg	VUS P	Parents	Died
29	F	HCM (Pompe Disease)	0.1	*GAA*	Splicing Glu888*	P P	Mother Father	Died
30	M	HCM (Danon Disease)	11.5	LAM	Splicing	VUS 3	None	Died
31	M	HCM (Pompe Disease)	0.6	*GAA*	Splicing	P	Parents	Died
32	M	HCM (Pompe Disease)	0.3	*GAA*	Arg168Pro Ser601Leu	LP LP	Mother Father	Died
33	M	HCM (Laing distalmyopathy)	13.2	*MYH7*	Splicing	VUS	Mother	Alive
34	M	DCM	0.2	*NEXN*	Pro609His	VUS	Mother	Alive
35	F	DCM	0.4	*LDB3* *MYBPC3* *MYH7*	Glu254Lys Ala1190Cys Ala728Val	VUS VUS VUS	Mother	Alive
36	M	DCM	8.6	*LMNA*	Glu290Lys	LP	None	Died
37	M	DCM	7.0	*ACTN2*	Met316Thr	VUS	Father	Alive
38	M	DCM	11.3	*PRDM16*	Asp628Asn	VUS	Mother	Alive
39	F	DCM	0.9	Undetected	/	/	/	Alive
40	M	LVNC	4.3	*MYPN* *MYH7*	Ser684Cys Arg904Leu	VUS LP	None None	Alive
41	F	LVNC	13.5	Undetected	/	/	/	Alive
42	F	LVNC	10.1	Undetected	/	/	/	Alive

*ACTN2* alpha-actinin-2, *DSP* desmoplakin, *GAA* acid alpha-glucosidase, *G6PC* glucose 6-phosphatase, DCM dilated cardiomyopathy, HCM hypertrophic cardiomyopathy, *LAM* lipoarabinomannan, *LDB3* Lim Domain Binding 3, *ILK* integrin linked kinase, *LMNA* lamin A/C, LVNC left ventricular noncompaction cardiomyopathy, *MYPN* myopalladin, *MYBPC3* cardiac myosin binding protein C, *MYH6* alpha-myosin heavy chain, *MYH7* beta-myosin heavy chain, *MYL2* myosin regulatory light chain-2, *MYPN* myopalladin, *NEXN* nexilin F-actin binding protein, *PKP2* plakophilin, *PRDM16* PR domain-containing 16, *RCM* restrictive cardiomyopathy, *RAF1* Raf-1 proto-oncogene, serine/threonine kinase, *TPM1* tropomyosin alpha-1 chain, *TNNI3* isoform of troponin I, *TTN* titin, *P* pathogenic, *LP* likely pathogenic, *VUS* variant uncertain significance

## Discussion

Cardiomyopathy is a group of myocardial disorders, mainly including DCM, HCM, LVNC and RCM, which contribute significantly to heart failure and cardiovascular mortality in children. Over recent years, there has been an increasing number of reports on the clinical features of cardiomyopathy in children, but these are mostly reported in North America and Europe. There are relatively few reports on cardiomyopathy in Asian populations, particularly in western China [1]. The current study involves a large clinical survey of childhood CM encompassing 317 patients in a Western Chinese populace. The purpose of this study was to describe the clinical characteristics and outcomes of these patients and to identify risk factors associated with death from cardiomyopathy.

DCM was the most common form of CM observed in this study, accounting for 40.1% of all patients, which is similar to the proportion of disease described in previous reports [2, 9, 10]. Consistent with prior studies [11], HCM was the second most common form of CM observed, accounting for 25.2% of all patients with CM. In the present study, LVNC accounted for 24.6% of pediatric cardiomyopathies, which was higher than reported in previous studies from America and Australia (5–10%) [12–14], indicating that its actual prevalence may be more frequent in Asian populations than previously recorded. Although the proportion of patients with RCM was higher (10.1%) than reported in previous studies (1.6–6.5%) [2, 15, 16], it remained the least common type of pediatric CM identified.

Age at diagnosis with CM differed significantly among the four groups. The majority of enrolled patients (66.6%, 211 patients) were less than 6 years of age at initial presentation, including 34.1% (72 patients) within the first year of life. The mean age at diagnosis of DCM was similar to that of RCM, which was significantly higher than the age at diagnosis of HCM and LVNC. It should be noted that DCM was previously reported to be commonly diagnosed in the first year of life [2, 17]. The older age at diagnosis observed in the present study may be attributed to the delay in diagnosis, resulting in a high mortality rate in young CM patients.

Our study demonstrated that the cumulative survival rates among the four CM groups were significantly different. Combining with analyses of overall survival and mortality, our results showed DCM patients had a better prognosis, whereas RCM patients demonstrated the worst outcome. We found high mortality was closely related to increased severity of clinical symptoms and HF. In this study, the five-year survival rate for DCM patients was 75.5%, compared with 72.6% in a previous study [2]. Meanwhile, the five-year survival rate of 67.3% in HCM patients was lower than previously reported [18, 19]. Moreover, our study showed a five-year survival rate of 74.1% in LVNC patients, compared with the rate of 80–91% reported by Ce Wang et al. and 52% reported by William Y Shi et al. [14, 20]. For RCM patients, the five-year survival rate of 51.1% was still lower than the rate of 64.6% in adult population, suggesting that patients who are diagnosed with RCM in childhood may have a worse prognosis than those diagnosed later in life [21].

In recent years, numerous studies have reported that the survival and death in CM are associated with several prognostic factors. In the DCM study by Cristina et al., mortality was higher in those with a family history of heart disease or sudden death and in those who required inotropic support during hospitalization [22]. The prognosis of DCM children was poor in those with HF, dilatation of the LV, and lower baseline left ventricular fractional shortening Z score [23]. Our univariate analysis also demonstrated that the classification of NYHA/Ross class III or IV, QTc interval, EF, FS, and moderate-severe TR affected prognosis. In addition, multivariate analysis showed that NYHA/Ross class III or IV and QTc interval were also associated with prognosis, consistent with findings in the adult CM population [24]. The predictive value of the coexistence of HF signs and other parameters has been described in patients with DCM [23, 25], suggesting that these parameters contribute to the diagnosis and prognosis of CM.

Patients with LVNC exhibited similar clinical features, echocardiographic characteristics and treatments, compared with DCM patients. However, LVNC is a distinct form of CM in pediatric patients, as indicated by differences in genetic basis and pathogenesis [26]. In our study, children with LVNC who developed clinical manifestations at an early age had worse outcomes than those with DCM, along with lower five- and ten-year survival rates. A previous study implied that congestive HF at diagnosis, infantile type and lower LVPW thickness Z-score were risk factors for death in all pediatric LVNC patients, while not the N/C ratio [20]. Interestingly, our report showed that N/C ratios of 2 or above was found in 50% of patients and that an elevated N/C ratio was a risk factor for death in the LVNC group. Additionally, QRS duration and elevated RVEDD were also risk factors in LVNC patients. In fact, the right ventricle and septum can be affected in LVNC, despite that noncompaction mostly affects the left ventricle. Our results showed that the group with elevated RVEDD also demonstrated poor prognosis, which differed from the result published on an adult population [27].

Unlike DCM and LVNC, HCM is characterized by hypertrophic ventricles and diastolic dysfunction. In our study, 15 patients (18.8%) presented systolic dysfunction with EF < 55%. A prior study demonstrated that the survival rate exhibited a significant decline within two years after diagnosis with a proportion of 71.2% patients at <1 year of age [5]. However, our findings did not show an association between the risk of death and age at diagnosis < 1 year or in other age groups. LVPW thickness, signs of HF, NYHA class ≥ III, LA size, and an LVEF of <60% have been reported as prognostic factors for death in HCM patients by Nasermoaddeli [28]. Based on a previous meta-analysis by Xia K, four major clinical risk factors for SCD in childhood HCM were: previous ventricular fibrillation (VF) or sustained ventricular tachycardia (SVT), unexplained syncope, non-SVT (NSVT), and extreme left ventricular hypertrophy (defined as a LV maximal wall thickness 30 mm [29]. Our findings supported that increased LVPW thickness is a risk indicator and preserved EF is a protective factor for survival. Signs of HF such as jugular venous distension and hepatomegaly, NYHA/Ross class III or IV, and pericardial effusion were risk indicators based on our univariate analysis, but not in our multivariate analysis. A pediatric scoring system HCM Risk-Kids tool and a novel risk prediction model for sudden cardiac death in childhood HCM have been proposed [6, 30]. The HCM-Risk-Kids model is available freely online (https://hcmriskkids.org) allowing clinicians to calculate individualized estimates of 5-year risk for their patients and Echocardiographic data on all CM patients at initial presentation are summarized perform an independent external validation of the model. However, this model was based on European children with HCM and there is a need for further external validation of whether this model is universal.

Investigation of prognostic factors for RCM is severely limited by its low morbidity, particularly in pediatric population. In this study, the worst prognosis was observed in 4 cases of RCM patients (12.5%) with systolic dysfunction (EF < 55%), reflecting the complex conditions in advanced HF. In children and young adults with RCM, especially those under 5 years of age, cardiomegaly and pulmonary venous congestion, elevated mitral valve Doppler E/e' ratio, and elevated left atrial pressure were correlated with adverse survival [31]. Moreover, in adult participants, male sex, each increase in NYHA functional class, left atrial diameter, advanced TR and lower LVEDD were reported to be inversely associated with survival by Ammash et al. and Jung Ae Hong et al. [32]. Our study showed that male sex, family history, orthopnoea, NYHA/Ross class III or IV, P-R interval, and LVEDD were indicators affecting survival rates based on univariate analysis. In addition, male, NYHA/Ross class III and reduced LVEDD were identified as independent risk factors by multivariate analysis. Nevertheless, reduced LVEDD – Z score was not correlated with adverse survival by multivariate analysis. These findings suggest that boys with increasing symptoms and signs of HF and restricted LV may have the worst prognosis. As described by Sherazi et al. [33], a smaller cavity in heart failure with preserved EF is more likely to develop enhanced passive chamber stiffness, and the small ventricle is often unable to adequately accept venous return, thereby worsening LV filling pressures, and such high filling pressures may worsen HF symptoms and increase the risk of death.

Gene mutations were found in 32 of 42 patients: 8 with RCM, 18 with HCM, 5 with DCM and 1 with LVNC. Only seven patients had a positive family history. A portion of children’s parents had low education level, which would limit family history inquiries. In our study, the parents of 19 individuals (45.2%) had positive findings in next-generation sequencing. This record was consistent with the previous single-center study [9]. Overall, the mutations of our cohort were similar to previous reports [1, 8]. *TNNI3* mutations are the most common gene variants in RCM. Three mutations have been described as pathogenic: 2 in *TNNI3* (Arg192Cys, Arg192His) and 1 in *PKP2* (Ala749Asp) [34–36]. Mutation at site 192 in *TNNI3* has been shown to be associated with Ca^2+^ hypersensitivity, leading to diastolic dysfunction and HF [37]. It is worth noting that a patient with *TTN* mutation presented with secondary RCM induced by Salih myopathy. In our study, all detected HCM patients carried gene mutations, and *MYH7* mutations were the most common gene variants detected in our HCM cases. One mutation in *MYH7* (Ala254Glu) was novel and localized in an important region of the gene. Additionally, Pompe disease with *GAA* mutations and Noonan syndrome with *RAF1* mutations are common secondary causes of HCM. The prognosis was poor in HCM patients with gene mutations, particularly in those younger than 1 year of age. Nearly half of the mutations were pathogenic and others were likely pathogenic or variants of uncertain significance, indicating more studies are needed to confirm their clinical significance and underlying mechanisms. Our findings support the hypothesis that patients with multigene mutations have a worse outcome than those with single-gene mutations or those with no detectable gene mutations.

## Limitations

The main limitation of the present study is the relatively small sample size recruited from a single medical center. This study may not represent the characteristics of all patients with CM. However, our results provide additional knowledge about the four cardiomyopathies. In this study, CM was diagnosed on the base of clinical and echocardiographic findings without cardiac catheterization or histological examination. In addition, despite the increasing use of genetic sequencing in cardiovascular disease, genetic analysis was only performed in a subset of patients, which may underestimate the association between genetic factors and cardiomyopathy. Finally, as a retrospective study, potential biases are likely and our results should be interpreted with caution.

## Conclusion

This study describes the clinical, echocardiographic and genetic characteristics and prognostic factors of patients with CM. DCM and RCM are the most common and least common types of CM, respectively. Genetic mutations in *TNNI3* and *MYH7* may play a key role in RCM and HCM. Our survival analysis showed differences between pediatric and adult populations, however, the prognosis of CM in children remains poor, especially in those with RCM. Some clinical features and parameters are helpful for evaluating prognosis. Although the predictors for the four cardiomyopathies are different, NYHA/Ross class III or IV should be considered as an indicator of adverse outcomes for the majority of patients with CM. However, accurate prediction of prognosis in children remains a great challenge. In the future, more nationwide large-scale studies and analyses including cardiac catheterization, biopsy, and genetic testing are needed to more accurately assess the disease profile and prognostic factors in children with CM.

### Supplementary Information

Below is the link to the electronic supplementary material.

Supplementary file1 (DOCX 110 KB)

## Data Availability

All data generated during review process will be available upon request from the corresponding author.
