# Supplementary material for: The clinical profile, genetic basis and survival of childhood cardiomyopathy: a single-center retrospective study
Source: Eur J Pediatr. 2024 Jan 2;183(3):1389–401. doi: 10.1007/s00431-023-05358-6 (PMC10951031; doi:10.1007/s00431-023-05358-6)

**Supplementary Information**

**Supplementary Figure legends**

**Supplementary Figure 1.** Breakdown of cardiomyopathy types in the present study. The cohort consisted of 127 dilated cardiomyopathy (DCM) cases (40.1%), 80 hypertrophic cardiomyopathy (HCM) cases (25.2%), 78 left ventricular noncompaction cardiomyopathy (LVNC) cases (24.6%) and 32 restrictive cardiomyopathy (RCM) cases (10.1%).

**Supplementary Figure 2.** Median ages at diagnosis with cardiomyopathy were significantly different with each other in four subgroups (*P* < 0.001). Dilated cardiomyopathy (DCM) group was diagnosed significantly older than those of hypertrophic cardiomyopathy (HCM) group (*P* < 0.001) and left ventricular noncompaction cardiomyopathy (LVNC) (*P* < 0.001). Restrictive cardiomyopathy (RCM) group was also diagnosed significantly older than those of hypertrophic cardiomyopathy (HCM) group (*P* = 0.006) and left ventricular noncompaction cardiomyopathy (LVNC) (*P* = 0.009) group.

**Supplementary Figure 1**


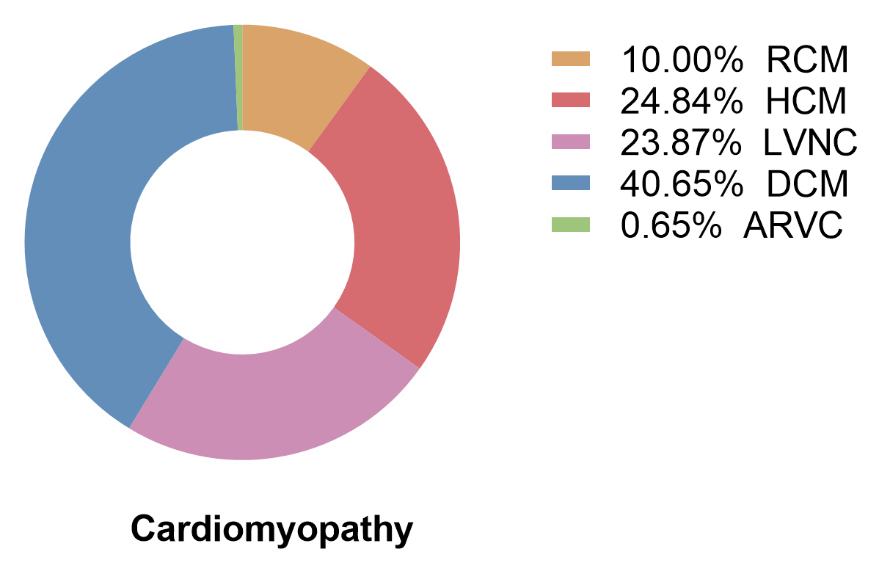


**Supplementary Figure 2**


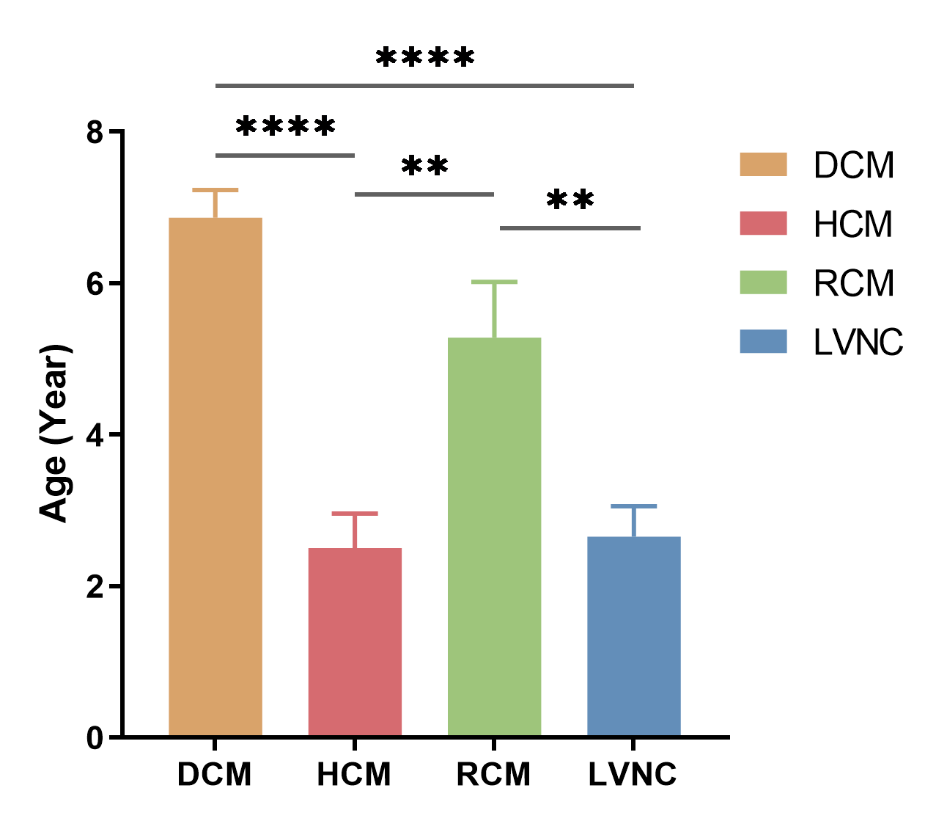

Supplement: Supplementary file 1 — Supplementary file1 (DOCX 110 KB) [file 431_2023_5358_MOESM1_ESM.docx]
